# 3D Timelapse Analysis of Muscle Satellite Cell Motility

**DOI:** 10.1002/stem.178

**Published:** 2009-10

**Authors:** Ashley L Siegel, Kevin Atchison, Kevin E Fisher, George E Davis, DDW Cornelison

**Affiliations:** a Division of Biology,University of MissouriColumbia, Missouri, USA; b Christopher H. Bond Life Sciences Center, University of MissouriColumbia, Missouri, USA; c Department of Medical Pharmacology and Physiology, and University of MissouriColumbia, Missouri, USA; d Department of Pathology and Anatomical Sciences, University of MissouriColumbia, Missouri, USA

**Keywords:** Adult stem cells, Cell migration, Muscle stem cells, Integrins, Satellite cells

## Abstract

Skeletal muscle repair and regeneration requires the activity of satellite cells, a population of myogenic stem cells scattered throughout the tissue and activated to proliferate and differentiate in response to myotrauma or disease. While it seems likely that satellite cells would need to navigate local muscle tissue to reach damaged areas, relatively little data on such motility exist, and most studies have been with immortalized cell lines. We find that primary satellite cells are significantly more motile than myoblast cell lines, and that adhesion to laminin promotes primary cell motility more than fourfold over other substrates. Using timelapse videomicroscopy to assess satellite cell motility on single living myofibers, we have identified a requirement for the laminin-binding integrin α7β1 in satellite cell motility, as well as a role for hepatocyte growth factor in promoting directional persistence. The extensive migratory behavior of satellite cells resident on muscle fibers suggests caution when determining, based on fixed specimens, whether adjacent cells are daughters from the same mother cell. We also observed more persistent long-term contact between individual satellite cells than has been previously supposed, potential cell-cell attractive and repulsive interactions, and migration between host myofibers. Based on such activity, we assayed for expression of “pathfinding” cues, and found that satellite cells express multiple guidance ligands and receptors. Together, these data suggest that satellite cell migration in vivo may be more extensive than currently thought, and could be regulated by combinations of signals, including adhesive haptotaxis, soluble factors, and guidance cues. Stem Cells *2009;27:2527–2538*

## INTRODUCTION

Skeletal muscle, which comprises up to 40% of human body mass, is a highly ordered, structurally stable tissue composed of differentiated, contractile myofibers arrayed within concentric sheaths of extracellular matrix and connective tissue. Very little turnover of muscle tissue occurs in vivo, except when muscle repair and/or regeneration is required to respond to overwork, trauma or disease. In such cases, a population of adult myogenic precursor cells, satellite cells, is required to provide a source of new myonuclei. In undamaged muscle, satellite cells are a rare, highly dispersed population of mitotically quiescent single cells, maintained in a niche outside the plasmalemma but beneath the basal lamina of host myofibers. This niche insulates the satellite cell from a majority of extracellular stimuli, allowing them to become “activated” only in specific conditions associated with damage or disease; known activating factors are currently limited to hepatocyte growth factor (HGF), nitric oxide (NO), and possibly tumor necrosis factor-alpha (TNF-α) (reviewed in [1,2]).

Once activated, satellite cells will emerge from beneath the basal lamina and proliferate extensively, establishing a large population of differentiation-competent myoblasts. These myoblasts will eventually differentiate and fuse either to one another or to existing myofibers to replace or repair damaged muscle. Interest in satellite and other muscle-derived stem cells has increased dramatically over the past several years, reflecting both the advent of new molecular and technical tools and increased realization of their clinical potential as vectors for cell and gene therapy. While myoblast transplantation as a therapy for muscular dystrophy was initially shown to be feasible in mice in 1989, albeit under conditions that would not be applicable to muscle disease therapy [3], subsequent efforts to engraft satellite cells or other myoblasts into host muscle have met with extremely limited success. Three major problems contributing to this lack of success are: 1) the majority of engrafted cells die within 3 days of injection; 2) if immunosuppression is not adequate, any surviving myoblasts are rejected within 2 weeks; and 3) those myoblasts that do survive do not migrate more than 200 μm away from the injection site (reviewed in [4]). This last hurdle, in particular, has proven difficult to overcome even as significant progress has been made on the other two. While current protocols can result in up to 30% dystrophin-positive fibers after treatment [5,6], they require a very large number of injections: the cited studies utilized 100 injections per square centimeter of muscle surface (up to 4,000 injections in a single individual) to treat highly restricted anatomical areas. These highly concentrated myoblast injections also lead to necrosis secondary to oxygen deprivation and toxic metabolites caused by such a large cell bolus [7].

It remains unclear to what extent cellular migration is involved in satellite cell-mediated regeneration. However, it seems plausible that given the isolation and relatively sparse distribution of satellite cells in uninjured tissue, accumulation of a large population of activated myoblasts at a site of focal injury would require directional motility. Early in vivo data is consistent with this hypothesis, suggesting that activated satellite cells may not only traverse the entire length of a myofiber, but will also migrate between fibers [8–11]. However, these studies were primarily descriptive rather than mechanistic, and few studies examining satellite cell motility have appeared in the literature since.

Asking specific questions about satellite cell migration in vivo is, like most other aspects of satellite cell physiology, complicated by the rarity and dispersion of satellite cells within the tissue. Conversely, in vitro experiments on large, purified satellite cell populations in adherent tissue culture remove any potential contribution of endogenous stimuli and substrates, and may introduce artifactual results, particularly when examining adhesion-based signals. In the most extreme case, immortalized myoblast cell lines are convenient and consistent, but are the furthest removed from an in vivo system (reviewed in [12]). To attempt to bridge the gap between these systems, we have developed a method to analyze primary satellite cells on their native migration substrate (the surface of a myofiber) by timelapse microscopy, in a programmable three-dimensional matrix. While it is not a true in vivo system, in that soluble and matrix-associated factors and forces that do not originate with either the satellite cells themselves or the single myofibers are absent, the relative advantages of this in vitro system are that it: 1) permits retention of the native adhesion and migration substrate for endogenous primary satellite cells, and 2) allows the establishment of reproducible, defined experimental conditions to test hypotheses and define genetic, protein, and morphologic changes in a temporally coordinated fashion. We can manipulate the availability of soluble factors and/or adhesion/guidance factors by supplementing the medium with exogenous factors or by adding blocking antibodies or pharmaceutical inhibitors. Multiple means of assessing the role of cell-intrinsic factors are also possible, including: 1) comparing cells from wild-type versus mutant mouse strains; 2) comparing cells from the same strain carrying conditional mutations in either the recombined or unrecombined state; 3) overexpressing factors of interest via viral transduction; or 4) knocking down factors of interest with siRNAs encoded in viral vectors.

We have used this system to establish that satellite cell motility is regulated by soluble factors such as HGF, as well as adhesion-based signaling between the myofiber external lamina and α7β1 integrin on the satellite plasma membrane. Additionally, we describe unexpected “behaviors” that suggest intriguing possibilities for satellite cell interactions with one another and their environments. In particular, the duration and closeness of associations between either sister cells or unrelated cells suggests that it is difficult to accurately interpret the cell lineage relationship of adjacent cells in static preparations. We also present novel data showing that primary satellite cells express a wide array of guidance receptors and ligands that are well known in the context of neuronal and endothelial cell migration, but which have not to date been examined in satellite cells. This suggests that active pathfinding by satellite cells may play a role in muscle regeneration and repair, and thus may constitute a new area of focus for both basic and therapeutic research.

## METHODS

### Satellite Cell Harvest and Culture

Satellite cells from B6D2 female mice (Jackson Labs, Bar Harbor, ME, http://www.jax.org) 80 to 130 days old were harvested and cultured according to our established protocol [13]. Briefly, muscle is dissected from the hind limbs, minced, digested in 400 U/mL collagenase type I (Worthington Biochemical, Lakewood, NJ, http://www.worthington-biochem.com), diluted in Ham's F-12 medium (Invitrogen, Carlsbad, CA, http://www.invitrogen.com), filtered, and collected by centrifugation. Cells are cultured on treated plates (Nunc, Rochester, NY, http://www.nuncbrand.com) coated with gelatin unless otherwise noted. Growth medium is Ham's F-12 (Gibco, Grand Island, NY, http://www.invitrogen.com), 15% horse serum (Equitech, Kerrville, TX, http://www.equitech-bio.com) and penicillin/streptomycin (Gibco) supplemented with 0.5 nM rhFGF-2. For the experiments described here, cells were examined at 4 days after harvest.

#### Reverse Transcriptase Polymerase Chain Reaction

Intron-spanning primers were written using PrimerSelect (DNAStar, Madison, WI, http://www.dnastar.com). Primer sequences and product lengths are listed in Supporting Table 1. Total RNA was harvested from primary adult myoblasts after 4 days in monoculture, and from proliferating MM14 cells or proliferating C2C12 cells, then reverse-transcribed to cDNA (SuperScript II, Invitrogen). 100 ng of each sample was used as template for simultaneous polymerase chain reactions (PCR).

#### Western Blotting

Lysates were prepared in Allen buffer from satellite cell-derived myoblasts after 4 days in monoculture and proliferating MM14 and C2C12 cells. 20 μg of each lysate was loaded onto a 4-12% gradient polyacrylamide gel (Invitrogen), transferred to polyvinylidene difluoride (PVDF) membranes, and blocked in Tris-buffered saline (TBS)-Tween containing 5% milk. Primary antibodies (BD Pharmingen, San Diego, CA, http://www.bdbiosciences.com/index_us.shtml) were incubated overnight at 4°C followed by horseradish peroxidase (HRP) conjugated secondary antibodies (Pierce, Rockford, IL, http://www.piercenet.com) for 45 minutes at room temperature. Chemiluminescent substrate (Pierce SuperSignal West) was detected with a LAS3000 imager (Fujifilm, Tokyo, Japan, http://www.fujifilm.com/products/life_science_systems/). All blots were stripped and reprobed with anti-IP90/calnexin (Abcam, Cambridge, U.K., http://www.abcam.com) to confirm equal loading; IP90/calnexin levels between cell types are equivalent per microgram of protein loaded.

#### Fluorescent Imaging

Cells in monoculture were plated on glass coverslips coated with 10 μg/ml gelatin, 10 μg/ml collagen, 10 μg/ml fibronectin (Bachem, Bubendorf, Switzerland, http://www.bachem.com) or 20 μg/ml laminin (Sigma, St. Louis, MO, http://www.sigmaaldrich.com) and allowed to adhere for 4 hours. Coverslips were fixed in cold 4% paraformaldehyde, incubated for 20 minutes with 1 μg/ml Alexa 488-labeled phalloidin (Invitrogen), mounted in Vectashield containing DAPI (Vector Labs, Burlingame, CA, http://www.vectorlabs.com), and imaged on an Olympus BX61 microscope (Olympus, Center Valley, PA, http://www.olympusamerica.com). Paraformaldehyde-fixed myofibers after 4 days of floating culture were stained for Robo1 (Abcam) which was detected with Alexa 594-conjugated goat anti-rabbit (Invitrogen). Images were collected in SlideBook (Intelligent Imaging Innovations, Denver, CO, http://www.intelligent-imaging.com).

#### Myofiber Harvest and Culture

Viable myofiber explants were produced according to our published techniques [13–15]. Briefly, muscle is dissected from the hind limbs, carefully separated from associated tissues, and digested in 400 U/mL collagenase type I (Worthington) diluted in Ham's F-12 medium (Invitrogen). When single fibers are liberated, they are manually picked with a pipette and cultured at 37°C and 5% CO_2_ in growth medium [Ham's F-12 (Gibco), 15% horse serum (Equitech), and penicillin/streptomycin (Gibco) supplemented with 0.5 nM rhFGF-2]. For standard 24–48 hour data collections, fibers are cultured for 24 hours before being repicked into 48-well plates for timelapse analysis.

#### Collagen Gel Culture and Timelapse Capture

3 to 5 myofibers are added to 200 μl of acid-extracted rat tail type I collagen (made in-house [16]; 2 mg/ml in growth medium) per well in 48-well plates (Corning, Lowell, PA, http://www.corning.com), and the collagen is allowed to rapidly polymerize at 37°C. The wells are then overlaid with growth medium containing supplements as appropriate [0.5 nM FGF-2, 80 ng/ml HGF (R&D Systems Inc., Minneapolis, MN, http://www.rndsystems.com), 200 ng/ml SDF-1 (R&D Systems), 1 uM lysophosphatidic acid (LPA) (Avanti Polar Lipids), 1 uM S1P (Avanti Polar Lipids, Alabaster, AL, http://www.avantilipids.com), or antibodies as listed below]. All conditions are represented by duplicate wells in every experiment. Multiple 10x fields are identified per well and marked for return; images are automatically collected from each field every 10 minutes using IPLab (Scanalytics, Rockville, MD, http://www.scanalytics.com).

#### Postimaging Analysis

Stacked images generated by IPLab (Scanalytics) are imported into MetaMorph (Axon Instruments/Molecular Devices Corp., Union City, CA, http://www.moleculardevices.com) and arranged in sequential order. Distance of migration is measured using digital pixel trace measurements. If a cell selected for tracking proliferates during the 24-hour collection period, one daughter cell is selected at random to continue the trace. Tortuosity of tracks was determined using Fractal5 [17,18], and is defined as the deviation from a correlated random walk, where 1 = a straight line and 2 = a path so tortuous as to cover the entire two-dimensional plane.

#### Neutralizing Antibody Treatment

Neutralizing sterile monoclonal antibodies to integrin chains were purchased from BioLegend (San Diego, CA, http://www.biolegend.com) (anti-α2, clone HMα2 [19]; anti-α4, clone 9C10(MFR4.B) [20]; anti-α4, clone R1-2 [21]; anti-α5, clone 5H10-27(MFR5) [20]; anti-α5, clone HMα5-1 [22]; anti-α6, clone GoH3 [23]; anti-αV, clone RMV-7 [24]; anti-β1, clone HMβ1-1 [25]; and anti-β2, clone M18/2 [26]) or MBL International (Nagoya, Japan, http://www.mblintl.com) (anti-α7, clone 6A11, [27]); all antibodies were tested for adhesion and blocking of substrate adhesion to confirm bioactivity. Collagen gel fiber preparations were treated with 25 μg/ml concentrations of individual antibodies except for anti-β1 which was at 50 μg/mL; when two antibodies were available to the same integrin chain, they were both added to achieve maximal blocking.

## RESULTS

### Myogenic Cell Adhesion and Motility on Purified Matrix Substrates Is Quantitatively and Qualitatively Distinct

The external lamina surrounding individual myofibers in vivo is primarily composed of type IV collagen and laminin [28], while the interstitial connective tissue contains type I, III, and V collagen and fibronectin [29]. In tissue culture, the choice of substrate can have dramatic effects on cell activity. Hauschka and Konigsberg reported in 1966 that collagen deposited by fibroblasts on tissue culture dishes promoted enhanced proliferation and differentiation of embryonic chick myoblasts [30]. Fibronectin and laminin have also been shown to have distinct effects on myoblast adhesion, morphology, and motility: while both will support adhesion, laminin specifically promotes myoblast locomotion while cells on fibronectin tend to organize vinculin-containing focal contacts and assemble α-actinin stress fibers [31]. To establish baseline measures for substrate effects on primary adult myoblasts, we adhered primary satellite cells to purified collagen, fibronectin, or laminin, then stained them with fluorescently-labeled phalloidin to visualize the actin cytoskeleton. We also included two commonly-used satellite cell-derived lines: C2C12 cells [32], which are the most commonly used myoblast cell line in adhesion/migration studies (as well as in most other applications), and MM14 myoblasts [33], which are less widely used but retain more of the morphological and molecular characteristics of primary satellite cells.

**Figure 1:**
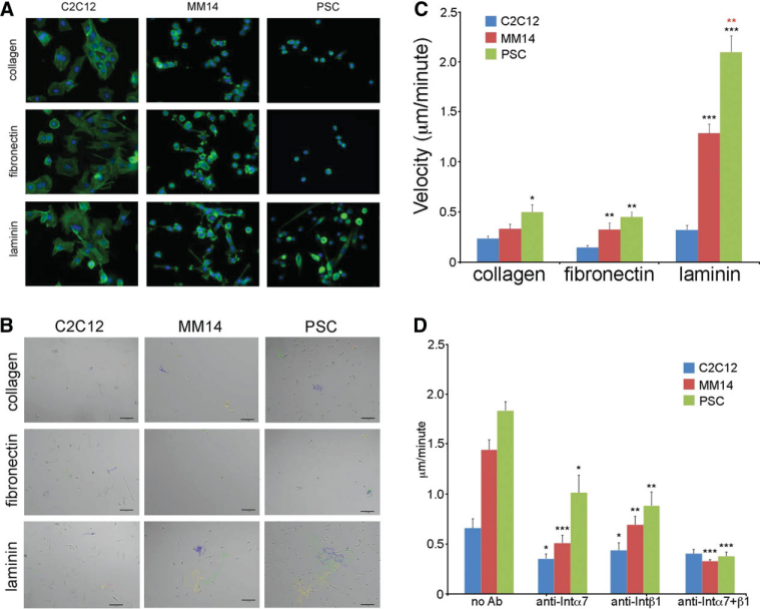
Primary satellite cells and cell lines display differential adhesion and migration on purified extracellular matrix (ECM) substrates; neutralization of α7β1 integrin blocks motility on laminin. **(A):** Fluorescence micrographs of f-actin (visualized with Alexa 488-phalloidin) and nuclei (visualized with DAPI) of each myogenic cell type plated on different ECM substrates. **(B):** Final images from representative videos of each cell type on collagen, fibronectin, and laminin; individual cell traces used to calculate average cellular velocity are highlighted in unique colors. Scale bar = 100 μm. Inclusive time on all videos is 14 hours. Movies 1b1, 1b2, and 1b3 correspond to the displayed laminin-adhered cell movies. Number of cells tracked per condition = 12. **(C):** Primary satellite cells are significantly more motile than cell lines on all substrates; MM14s and primary satellite cells are significantly more motile on laminin than other substrates. Number of cells tracked per condition = 12. **(D):** Inhibition of integrin α7, integrin β1, or both by neutralizing antibody significantly decreases motility of MM14 and primary satellite cells on laminin. * = *p* < .05; ** = *p* < .01; *** = *p* < .001. Number of cells tracked per condition = 12. Abbreviation: PSC, primary satellite cells.

We find that C2C12 cells maintained their characteristic flattened morphology on all three substrates with extensive f-actin networks and extensions, while MM14 cells and primary satellite cells are rounded when adhered to either collagen or fibronectin, but become polarized and elongated when adherent to laminin (Fig. 1A). Satellite cells and myogenic cell lines seeded on purified extracellular matrix (ECM) components also display differential motility, consistent with previous descriptions of MM14 locomotion in vitro [31]. Cells plated on collagen I adhere and retain a characteristic round morphology, but are not motile (Fig. 1B). Cells plated on fibronectin adhere minimally and remain very transiently attached (Fig. 1B). However, cells plated on laminin are strongly adherent, take on a bipolar, flattened morphology, extend cellular processes, and in the case of MM14 cells and primary satellite cells, have significantly greater mobility (Fig. 1B; movies 1b1, 1b2, and 1b3). We observed clear differences in motility between primary cells and cell lines: on laminin, primary cells are more than sixfold more motile than C2C12 cells, and 1.6-fold more motile than MM14s (Fig. 1C).

The cellular receptors for ECM components including laminin and fibronectin are integrins, a family of type I transmembrane glycoproteins found only in metazoans (reviewed in [34]) that combine one of 18 α chains and one of 8 β chains into one of 24 permitted pairings to form functional heterodimers with unique substrate affinities. Embryonic/fetal myoblasts and cell lines derived from embryonic skeletal muscle have previously been shown to express α4, α5, α6, α7, αV, and β1, while adult skeletal muscle expresses α5β1, α6β1, and α7β1, primarily at myotendinous and neuromuscular junctions (reviewed in [35]). α5β1 integrin is a fibronectin receptor, whereas α6β1 and α7β1 show strong specificity for laminin (reviewed in [36]). The deletion phenotypes of α5 and α7 integrin both include muscular dystrophy, suggesting a critical role for signaling through these subunits (reviewed in [34]), and α6 integrin is upregulated in regenerating *dy/dy* dystrophic mice [37]. To assess the role of specific integrin chains in 2D motility, we treated each cell type/substrate with neutralizing antibodies directed against integrins α4, α5, α6, α7, β1, or β2. Blocking α7 integrin, β1 integrin, or both together additively and significantly decreased the velocity of MM14 cells and primary satellite cells (Fig. 1D); no other treatment/condition produced a significant change in motility (data not shown). This is consistent with a role for binding to laminin through the integrin α7β1 receptor in satellite cell motility.

To determine if the collagen matrix used in the 3D assays described below could affect satellite cell motility independently of supplementation, primary cells were plated on laminin as described above and half of the wells were overlaid with collagen; cells were then tracked for 24 hours. We noted no significant differences in cell morphology or motility (data not shown).

### Primary Satellite Cells and Myogenic Cell Lines Differentially Express a Wide Variety of Integrin Chains

**Figure 2:**
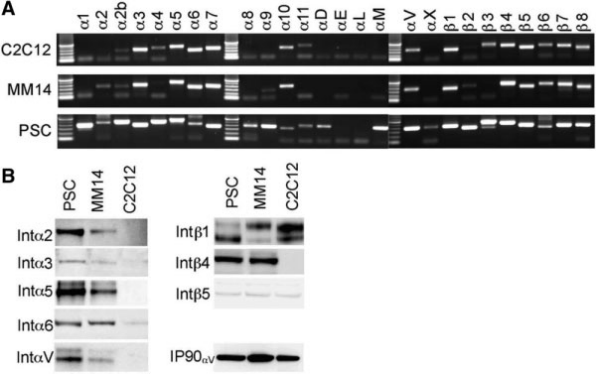
Primary satellite cells express a larger number of integrin mRNAs than cell lines: C2C12 cells express some integrin proteins differently compared to primary cells and MM14 cells. **(A):** Expression of integrin mRNAs in C2C12, MM14, and primary satellite cell-derived myoblasts; identical samples were processed simultaneously. Unlike immortalized cell lines, primary cells are positive for most or all known integrin chains (some samples of primary cells were positive for expression of integrins αE and αL, but not all were, including the one shown here). Intron-spanning primer sequences may be found in Supporting Table 1. **(B):** Expression of integrin protein in primary satellite cells (PSC), MM14, and C2C12 cells: all blots were stripped and reprobed with IP90 (calnexin) as a loading control; shown is anti-αV after stripping and reprobing.

The contribution of satellite cells to studies of adult muscle integrin expression is likely to be undetectable, since they make up such a small fraction of the total muscle mass; no comprehensive survey of adult satellite cell integrin expression has yet been performed. To determine the repertoire of integrin chains present in adult myoblasts, we assayed primary satellite cells, MM14 cells, and C2C12 cells for integrin chain expression. By RT-PCR we can detect surprisingly broad integrin expression in satellite cells: all or most known integrins (αE and/or αL are not detected in some samples) can be detected in primary satellite cells, while a smaller subset are detected in myogenic cell lines (Fig. 2A). We extended this result with Western blotting using chain-specific antibodies: while primary cells express slightly more integrin protein than MM14 cells, C2C12 cells possess significantly less than would be expected, and both cell lines express more of a higher molecular weight isoform of integrin β1 (potentially integrin β1D, an isoform associated with the formation and stabilization of focal adhesions [38]) than primary cells (Fig. 2B). There are also apparent discrepancies between the relative amounts of integrin chain mRNA expression and protein expression, especially in C2C12 cells: while some chains, such as α2 integrin, show lower levels of expression of both transcript and protein, several others show robust bands by RT-PCR and minimal or no signal by Western blot. We speculate that this could be due to the standard practice of culturing C2C12 cells on plastic, with no available physiological substrate, however this is untested. These results suggest that satellite cells may have greater flexibility in their potential for attachment and interaction with the ECM than was previously appreciated.

### Satellite Cell Motility on the Exterior Surface of the Host Myofiber Includes Unexpected Cellular Activities: “You Can Observe a Lot by Watching”

**Figure 3:**
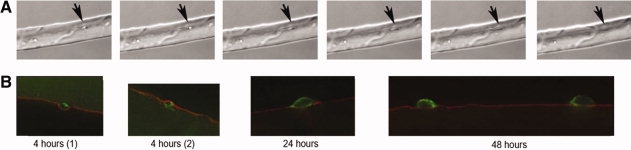
Fiber-associated satellite cells are sublaminar immediately after harvest, but emerge from the sublaminar niche and adhere to the exterior of the myofiber after activation. **(A):** Reference still images extracted from movie 3a. A satellite cell 12 hours after fiber harvest exits from a depression in the myofiber (its presumptive quiescent location, indicated by arrowhead). Movie 3a: inclusive time 12 to 21 hours after activation; stills taken at 12:00, 12:30; 13:00; 13:10; 13:20; and 14:00 hours. **(B):** Reference still images extracted from movie 3b containing Z-scans and/or volume renderings of satellite cells at 4, 24, and 48 hours after fiber harvest, stained with anti-laminin (red) and anti-syndecan-4 (green). Although cells are sublaminar at 4 hours, they appear to have left the sublaminar niche by 24 hours, and are clearly outside the basal lamina by 48 hours.

Quiescent satellite cells reside between the sarcolemma and the exterior lamina of the host myofiber; after activation, they move from beneath the lamina to the surface of the myofiber. We observed that satellite cells in 3D fiber culture exit their sublaminal niche as early as 12 hours after synchronous activation by fiber harvest (Fig. 3 and movie 3a). We confirmed that satellite cell movement after activation is on the exterior of the myofiber by costaining isolated fibers before and after satellite cells have become visible outside the basal lamina with anti-laminin (to delineate the myofiber's exterior surface) and anti-syndecan-4 (to identify the satellite cell plasma membrane). While satellite cells are still beneath the laminin sheath at 4 hours after harvest, at 24 or 48 hours, they are instead adhered to the exterior of the myofiber (Fig. 3 and movie 3b).

**Figure 4:**
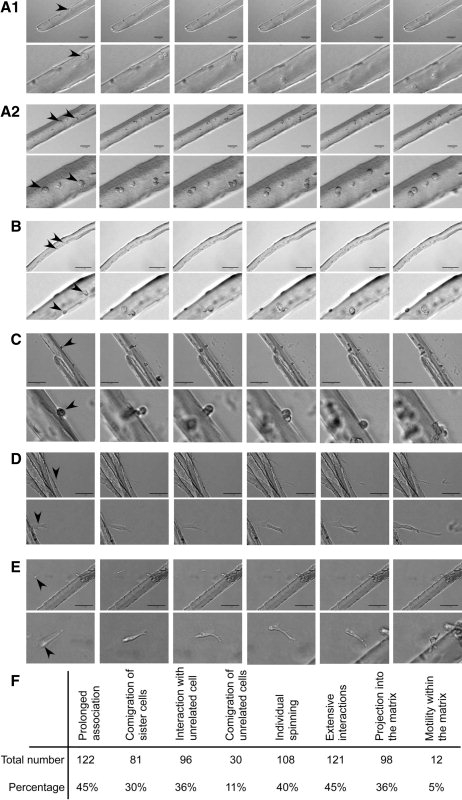
Fiber-associated satellite cells exhibit multiple novel “behaviors.” Figures are reference stills taken from the associated movies; magnified regions are shown below full-scale images. **(A):** Two movies showing recently-divided sister cells remaining closely associated for extended periods while moving along the fiber. Movie 4a1: Inclusive time 24 to 48 hours after activation, stills taken at 28:30; 28:40; 30:10; 32:10; 33:20; 45:40 hours. Movie 4a2: inclusive time 24 to 51 hours after activation, stills taken at 30:00; 30:30; 32:20; 34:30; 35:20; 48:50 hours. **(B):** Beginning at 12 hours (prior to any cell division), two presumably unrelated satellite cells migrate toward each other, associate, and remain closely associated for an extended period. Movie 4b: inclusive time 12 to 36 hours after activation, stills taken at 21:40; 25:20; 25:50; 26:30; 29:20; 36:00 hours. **(C):** Multiple satellite cells remain very closely associated for an extended time, interacting extensively but not fusing. Movie 4c: Inclusive time 48 to 72 hours after activation, stills taken at 48:00; 54:20; 59:20; 61:30; 69:10; 72:00 hours. Note that while some apparent “planar” cell divisions can be (as in still 2) other cell associations that appear to be planar cell divisions are almost certainly not (as in still 3). **(D):** A cell repeatedly extends protrusions into the collagen matrix, then moves off the fiber; another cell moves out along the first cell, divides, and the daughter cells continue in opposite directions. Movie 4d: inclusive time 48 to 72 hours after activation; stills taken at 52:20; 63:20; 65:20; 66:40; 67:10; 68:00 hours. **(E):** A cell in the collagen matrix moves parallel to a fiber, turns, and migrates onto the fiber. Movie 4e: Inclusive time 62 to 70 hours after activation, stills taken at 64:00; 65:10; 66:50; 67:20; 68:00; 68:50 hours. Note: area of magnified image is moved from frame to frame. **(F):** Relative frequencies of described activities in FGF-2-treated cultures from 24 to 48 hours after harvest. See Supporting Table 2, supporting movie Information, and additional Quicktime movies for more detailed information.

After activation, satellite cells begin to proliferate and move along the myofiber. While making qualitatitive observations, we noted several activities and interactions that had not previously been described. These include prolonged association and co-migration of sister cells after division (Fig. 4 and movie 4a1 and 2), as well as of unrelated cells (Fig. 4 and movie 4b). Some cells participate in extended, dynamic interactions (Fig. 4 and movie 4c). Extension of pseudopodia in opposing directions produces the characteristic bipolar spindle formations of muscle cells, and we observe both bipolar and unipolar membrane extensions in migrating satellite cells. The cell body remains stable until one pseudopod releases, then the cell body moves rapidly toward the still attached pseudopod, resulting in incremental movement along the fiber. Some cells also move through the collagen matrix, in which case we frequently observed ruffled lamellae at the distal edges of the leading pseudopods that appeared to be sampling the surrounding environment (Fig. 4 and movies 4d and 4e). While no labeling has been done to confirm the identity of these cells as satellite cells, and we therefore cannot rule out the possibility that they represent a different cell type, this would be consistent with in vivo data suggesting that satellite cells are capable of moving from one fiber to another [39].

We tabulated the frequency of each activity among 270 tracked cells in fiber cultures treated with FGF-2, and find that they occur with varying but relatively high frequency (Fig. 4F). An expanded version of this analysis may be found in Supporting Table 2, and full-length movies used to generate this table may be accessed at http://cornelisond.biology.missouri.edu/Cornelison_lab/movies/index.html. See the Supporting Movie Information file for complete descriptions of criteria used to score cell activities.

### Addition of Hepatocyte Growth Factor to the 3D Collagen Matrix Increases Directional Motility Along the Myofiber

Motility requires sequentially establishing, then releasing, adhesive contacts between areas of the cell membrane to matrix substrates; soluble motogens are frequently the stimulus for adhesion receptor and cytoskeletal modifications that allow such transient adhesions. Soluble factors affecting myoblast motility include numerous well-studied cytokines, chemokines, and growth factors that would be expected to be available in the context of acute muscle damage. Hepatocyte growth factor (HGF), originally referred to as “scatter factor” because of its pro-motogenic effect on epithelial cells [40,41], has been shown to be essential for migration of somitic myoblasts to the developing limb bud [42,43]. It has been shown to be a potent motogenic factor for numerous primary cell types [44–46] including satellite cells [39,47]. HGF signaling through its high-affinity receptor, c-met, results in activation of the Ras-ERK MAP kinase cascade and the phosphatidylinositol 3-kinase (PI3K) pathway, both of which are independently required for cell migration [48–50]. Recent data suggest that these pathways converge in myoblasts to promote formation of both filamentous and branched actin structures (in filopodia and lamellopodia, respectively) by the actin-related protein 2/3 (Arp2/3) complex [51]. The small chemokine CXCL12, also referred to as stromal cell derived factor-1 (SDF-1), is the sole ligand for CXCR4, a seven-transmembrane pass receptor which has also been reported as a marker for quiescent and activated satellite cells [52]. During development, SDF-1 is a chemoattractant for emigrating myoblasts [53]. SDF-1 is secreted by resident fibroblasts in skeletal muscle and increases during injury [52] and would therefore be available to influence satellite cell activity. In addition to growth factors and cytokines, signaling lipids have been reported to stimulate migration, survival, and proliferation in different cell types [54]; significant effects have been observed using both S1P (sphingosine 1-phosphate) and LPA (lysophosphatidic acid).

**Figure 5:**
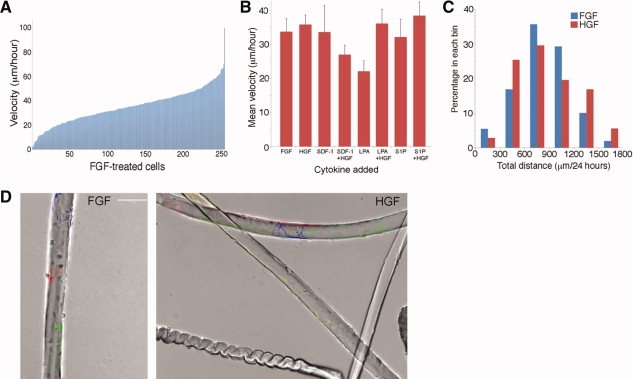
Exogenous cytokines can influence satellite cell motility: hepatocyte growth factor (HGF) increases directional persistence and decreases tortuosity. **(A):** Motility of 255 individual FGF-2 treated, fiber-associated satellite cells from 24 to 48 hours after isolation, arranged by increasing average velocity. All cells tested in all conditions showed displacement of at least 50 μm over 24 hours. **(B):** Average velocity in the presence of FGF-2 (control), HGF, lysophosphatidic acid (LPA), S1P, and pairwise combinations of factors. Number of cells tracked per condition ranged from 11 to 35. No treatment produced a statistically significant change in cell velocity from FGF-2 treatment. **(C):** Percent of FGF-2 or HGF-treated cells binned by total motility in 300 μm increments. A larger fraction of HGF-treated cells populate the high-motility bins. **(D):** Stills showing the final frames of movies 5b1 and 5b2. Tracings of fiber-associated satellite cells from the same mouse, with similar total velocity, over an identical time period, at an identical scale, treated with either FGF-2 or HGF illustrate that cells treated with HGF tend to have increased directional persistence. Inclusive time of both movies was 24 to 48 hours. **(E):** Comparison of tortuosity between tracks of FGF2-treated cells and HGF-treated cells: HGF treatment significantly decreases cell turning (*p* < .001). Number of cells tracked is 270 for FGF, 70 for HGF.

FGF-2, HGF, SDF-1, S1P, and LPA were added at uniform concentrations across the entire well to the collagen matrix, singly or in combination, at bioactive concentrations [39,52,54,55]. In all conditions tested, all cells moved a minimum distance of 50 μm from their site of origin. The great majority were significantly more motile (traveling up to 2,500 μm in 24 hours), which is significantly longer than the maximum reported distance traveled by exogenous satellite cells 6 weeks after experimental engraftment (200 μm) [56] but consistent with reports of extensive endogenous satellite cell migration in vivo [8,10]. Figure 5A shows average motility from 24 to 48 hours after harvest for 255 individual fiber-associated satellite cells treated with FGF-2. When exogenous HGF or other potential motogens were included in the polymerized collagen matrix, they did not significantly affect the mean distance traveled (Fig. 5B), although longer-migrating cells are more common with HGF treatment (Fig. 5C). However, when tracings of individual satellite cell movement are compared, HGF treatment leads to striking increases in linear displacement compared to FGF-treated cells (Fig. 5D and movies 5d1 and 5d2). When the tortuosity of cell tracks (rate and angle of turning) is compared between FGF2- and HGF-treated cells, HGF was found to produce significantly straighter paths as well (Fig. 5E). In the format used here (uniform addition to the culture), no other conditions led to significant changes in motility, although there were significant differential effects on cell proliferation (data not shown). While it is possible that a gradient or point source of cytokines would elicit directional or proximity-based responses, such experiments are not currently feasible to analyze in this system.

### α7β1 Integrin Neutralization Specifically Inhibits Satellite Cell Motility on the Surface of Myofibers

**Figure 6:**
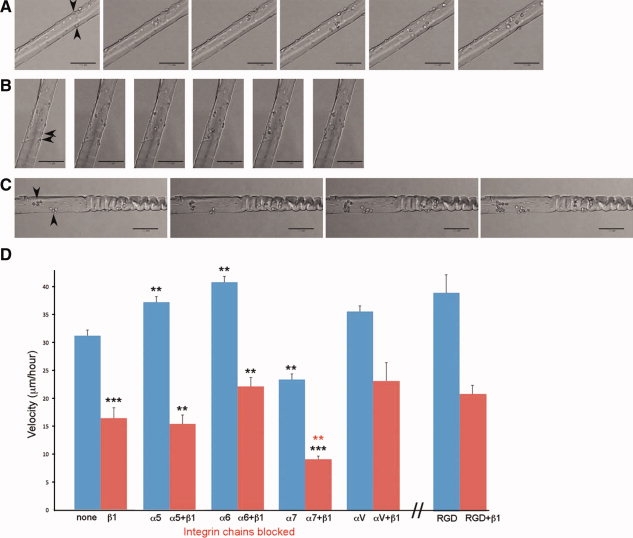
Blocking α7β1 integrin with neutralizing antibodies specifically inhibits satellite cell motility on myofibers. **(A, B, C):** figures are still images extracted from movies 6a, b, c.; inclusive time for all movies is 32 to 48 hours after activation. **(A):** Cells in 3D myofiber culture treated with neutralizing antibody to α7 integrin, stills taken at 32:00; 33:40; 36:00; 40:40; 43:00, and 48:00 hours. **(B):** Cells in 3D myofiber culture treated with neutralizing antibody to β1 integrin, stills taken at 32:00; 34:50; 36:00; 41:40; 43:00, and 48:00 hours. **(C):** Cells in 3D myofiber culture treated with neutralizing antibodies to α7 and β1 integrins, stills taken at 32:00; 36:00, 41:20; and 48:00 hours. Note that cell proliferation and cell-cell interactions are not blocked by integrin neutralization. **(D):** Quantitative representation of satellite cell motility in 3D myofiber culture after treatment with neutralizing antibody to α6 integrin, α7 integrin, αV integrin, β1 integrin, cyclic RGD, and pairwise combinations. Neutralization of α7 and β1 integrin, but neither neutralization of α6 or αV nor cyclic RGD, decreases satellite cell motility. * = *p* < .05; ** = *p* < .01; *** = *p* < .001. Number of cells tracked per condition ranged from 6 to 35.

We demonstrated in Figure 1 that binding to laminin via α7β1 integrin appears to be critical for satellite cell motility in monoculture. To determine whether this is an accurate reflection of motility on the host myofiber, we added neutralizing antibodies to several integrin α chains, individually and in combination with a neutralizing antibody directed against β1 integrin. Of the conditions tested, only neutralization of α7 (Fig. 6 and movie 6a), β1 (Fig. 6 and movie 6b), or both together (Fig. 6 and movie 6c) had a negative effect on satellite cell motility in 3D myofiber culture (Fig. 6D); the effects of blocking both are also additive. This is in contrast to blockade of either α6 (also a specific laminin receptor), α5 (a fibronectin receptor), or αV, all of which actually *increased* satellite cell velocity. Blocking antibodies to α2, α4, and β2 also do not decrease satellite motility on myofibers, although all antibodies tested successfully blocked cell adhesion in monoculture as appropriate (data not shown). In addition, treatment with cyclic RGD peptides, which would block multiple integrins including αVβ3, αVβ1, αVβ5, αVβ6, αVβ8, and αIIbβ3, and which has been shown to block human satellite cell motility in monoculture [57], does not inhibit satellite cell migration on the myofiber. One could speculate that the cell-matrix interactions mediated by integrins other than α7β1 may be involved in adhesive interactions with the myofiber matrix, such that their inhibition permits an increase in cell velocity along the myofiber. Although motility is dramatically decreased, neither cell adhesion to the myofiber, cell proliferation, or cell-cell interactions appear to be affected by α7β1integrin blockade. These data support the conclusion that the primary in vivo substrate for satellite cell migration is the exterior lamina of the host myofiber, engaged by the α7β1 laminin receptor on satellite cells.

### Satellite Cells Possess Multiple Signaling Pathways Associated with Extracellular Guidance Cues

**Figure 7:**
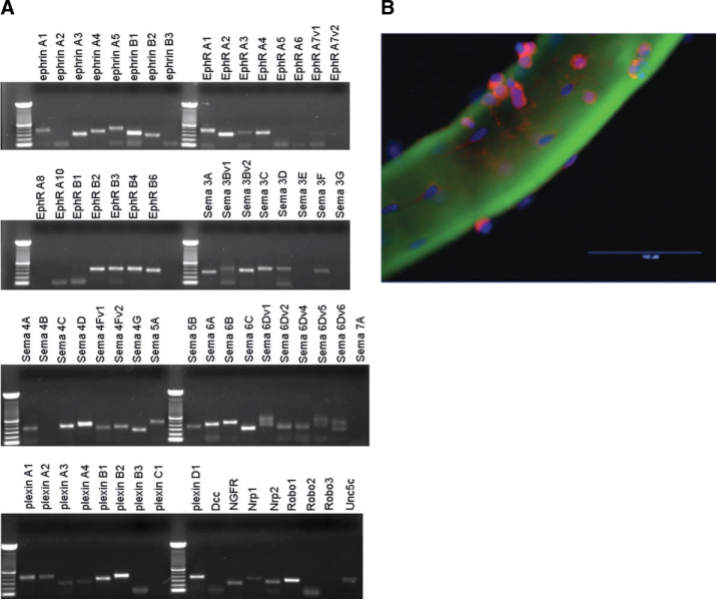
Satellite cell-derived myoblasts express mRNAs for multiple guidance receptors. **(A):** Reverse transcriptase polymerase chain reaction for gene products associated with migration guidance receptors; multiple classes of guidance receptor are represented. Intron-spanning primer sequences can be found in Supporting Table 1. **(B):** Immunohistochemistry for Robo1, a transmembrane guidance molecule receptor (Abcam); expression is exclusive to the satellite cells and appears localized to the satellite cell membrane contacting the myofiber surface. Scale bar = 100 μm. Abbreviation: HGF, hepatocyte growth factor.

A significant body of knowledge currently exists regarding chemotactic and contact-based attractive and repulsive signaling pathways and components, particularly in the context of migrating neurons, neural crest, and vasculogenic cells. Several key pairs of signaling molecules have been characterized as regulating guided cell migration, including semaphorins and plexins, Ephs and ephrins, netrins and Dcc/Unc5 family members, and Robos and Slits (reviewed in [58–62]). To assess whether satellite cell motility might involve responses to specific migration cues, we used RT-PCR and immunohistochemistry to evaluate their expression of guidance receptors and ligands. Surprisingly, we found that multiple guidance factors are strongly represented in activated satellite cells: mRNAs encoding members of all four major classes of guidance receptors were detected, as well as ephrin and semaphorin ligands (Fig. 7A). Immunostaining of fiber-associated satellite cells confirmed that guidance receptor expression is specific to satellite cells: Figure 7B shows localization of Robo1 on fiber-associated satellite cells, but not on the fiber itself.

## DISCUSSION

As the resident stem cell of skeletal muscle, satellite cells are of interest both as activatable adult stem cells and as potential vectors for cell or gene therapy. Current work on the “life cycle” of satellite cells after injury is generally focused on the molecular mechanisms mediating activation from the quiescent state, proliferation to form a pool of myoblasts sufficient to replace myonuclei lost to damage or disease, and differentiation and fusion to regenerate the injured myofibers. The generation (and re-generation) of quiescent satellite cells remains the focus of significant research interest as well. However, inquiry into a potential role for satellite cell motility or migration in vivo is comparatively scarce. This is largely due to technical difficulties in visualizing satellite cells dynamically within the muscle tissue, but may also be influenced by the observations that individual satellite cells are sufficiently proliferative to be able to fully replace damaged muscle locally [63] as well as the comparatively minimal motility observed in vitro, particularly in experiments using C2C12 cells as a model. In this report, we used a 3D timelapse culture system to assess the movement of primary satellite cells on their host myofibers and to begin to characterize the molecular and cellular mediators of satellite cell motility.

When we compared primary mouse satellite cells with the two most frequently used myogenic cell lines, C2C12 and MM14, in monoculture on purified matrix components including collagen, fibronectin, and laminin, we observed cell type-specific differential morphology, adhesion, and motility on different substrates. Most striking were the differences in motility between primary cells and C2C12 cells, which are frequently used as a satellite cell model in migration and adhesion assays. Both qualitative differences in integrin chain expression and quantitative differences in the relative amounts of expression were observed between primary adult myoblasts and C2C12 cells. This leads us to hypothesize that satellite cells may have the ability to interact with a much broader range of substrates than was previously considered, potentially including unfolded proteins, cell adhesion molecules such as ICAM, VCAM, and MadCAM, as well as ECM components not commonly studied in the context of skeletal muscle such as fibrinogen, von Willebrand's factor, vitronectin, thrombospondin, and osteopontin. This may have important implications for understanding satellite cell adhesion and motility in the context of damaged and regenerating muscle in vivo. The lack of many of these more uncommon integrins in muscle cell lines, together with the increased motility of primary cells, suggests that studies performed on cell lines may underestimate aspects of satellite cell migration, particularly in vivo.

In the novel context of this 3D timelapse system, we made qualitative observations of previously unreported satellite cell behaviors. In particular, the tendency of some sister cells to maintain physical contact for long periods of time after division and across long migration distances raises a caveat for interpretations of differential staining among adjacent cells in fixed fiber preparations. Maintenance of stem/progenitor cells by asymmetric cell division is an important and well-established paradigm in adult stem cells, including satellite cells. Asymmetric distribution of cell-surface proteins [64], expression of nuclear transcription factors [65], or segregation of parental DNA strands [66,67] in muscle satellite cells can be used to identify cells generating daughters with different identities, such as a progenitor cell and a more committed myogenic cell. When observed in the context of the host myofiber in vivo, these asymmetric divisions also appear to occur in a planar orientation [65]. Particularly given that such methods exist to validate *bona fide* asymmetric cell divisions, our data suggest that more rigorous criteria than the appearance of differently staining, adjacent cells in static preparations should be applied to verify such an event. We were also intrigued to note that some cells detach from their host fiber and migrate through the collagen matrix, in many cases moving in the direction of other myofibers embedded in the same well. These cells can be detected re-joining a fiber, after which they change their morphology from an elongated, polar appearance to the more rounded shape more often seen in fiber-associated cells. It is worth noting that these behaviors have all been observed during a relatively short timeframe: in our hands, satellite cells are activated as a consequence of fiber harvest, and most cultures were analyzed during the 24–48 hour period after fiber isolation.

It is important to note that while we have preserved one critical aspect of the in vivo environment (the interaction of a primary cell with a physiological substrate) numerous other known and unknown components of a true in vivo system are absent. The influence of extracellular matrix molecules associated with the areas between myofibers, of adjacent myofibers themselves, and of soluble factors that are not replicated in our system represent a significant caveat in contemplating whether these activities and behaviors occur in vivo, with what frequency, and whether they differ between, for example, healthy and dystrophic muscle regeneration. In addition, it would be intriguing to assess whether the observed behavioral heterogeneity in vitro is correlated to functional and phenotypical heterogeneity in vivo. As in vivo imaging capabilities increase, ideally it will eventually be possible to replicate these studies in situ.

When we stimulated fiber-associated satellite cells with soluble factors that have previously been described as motogenic factors for myoblasts in other contexts, we were surprised to observe that there were no significant effects on cell velocity, although there were changes in cell proliferation (data not shown). This may be due to the release of saturating amounts of cytokines from the myofibers and/or satellite cells present in the culture, the stage of the satellite cells being examined, or an effect of adhesion to the fiber rather than a tissue culture substrate. In particular, the potential contribution of soluble factors released from the myofibers or changes in their adhesive properties due to the culture conditions or changes in myofiber integrity (including fiber death or semihypercontraction) should not be discounted. However, while it did not significantly increase total velocity, HGF alone did produce a striking increase in cell displacement: cells treated with HGF traveled greater distances from their origin and moved more directly, compared to cells stimulated with FGF or other cytokines. In light of the critical roles in satellite cell activation and proliferation already associated with HGF signaling, it is intriguing to postulate a role for HGF in facilitating long-distance migration of activated satellite cells as well.

Distinct from simple motility, directed migration and pathfinding are mediated by the activity of cell surface guidance receptors. Signaling through these diverse receptors results in either attraction or, more frequently, repulsion, organizing cytoskeletal changes at the leading edge of motile cells. While a large and detailed body of literature is available in the context of other cell types, particularly neural crest, neurons, and endothelial cells, comparatively little has been published in muscle and there has been no suggestion that satellite cells are capable of directed guidance. While they have not been examined in the context of myoblast migration, several members of this class have been shown to be necessary for myogenic differentiation in vitro [68,69]. This may reflect a requirement for cell migration to aggregate and fuse to form myotubes [69,70], or a role in contact-mediated signaling distinct from migration guidance. After observing the morphological and directional changes that satellite cells display in single fiber culture, including apparent sampling and pseudopodial movement grossly similar to neuronal growth cones, we assayed primary satellite cells for expression of cell guidance receptors. Surprisingly, we found that all major classes of guidance receptors are represented, as well as two classes of guidance ligand, supporting the possibility that satellite cells may in fact actively migrate, presumably toward a site of local injury. The majority of the guidance receptors whose expression we detected on primary satellite cells usually elicit repulsive interactions when they bind their ligand (reviewed in [71]), suggesting that satellite cells might be maintained in a motile, transiently adherent state through their interactions with immobilized guidance cues.

We propose a model in which soluble motogens such as HGF, released by damaged areas of the myofiber, promote satellite cell motility, while repulsive interactions with comparatively intact areas of the myofiber maintain cell motility in the absence of injury. Injured myofibers, and/or immune cells recruited to the site of the injury, could be the source of an ad hoc chemoattractant gradient, while repulsive guidance cues would be lost on damaged areas of the fiber lamina. Future experiments in programmed matrices will test this model. In addition to suggesting an intriguing new functional process in these adult stem cells, these results may have important clinical implications for the design and execution of satellite cell-based therapies.
