# Supplementary material for: 3D Timelapse Analysis of Muscle Satellite Cell Motility
Source: Stem Cells. 2009 Oct;27(10):2527–38. doi: 10.1002/stem.178 (PMC2798070; doi:10.1002/stem.178)
Supplement: Supplementary file 1 [file stem0027-2527-SD1.pdf]

Gene Symbol	Acc #	Forward	Location	Reverse	Location	Product Size
Itga1	NM_001033228	CCGGCCAGGTCGTCATCTACAAG	1815-1837	CCAGGGGAGCTCCAATCACGAC	2216-2195	402
Itga2	NM_008396	GCGCCCGGGTGCTACAAAAG	909-928	AAAAGCCCCCACTGCACCTAACA	1305-1283	397
Itga2b	NM_010575	CCGCCCGGGTACCCTTTTGTG	882-902	CTTGCGGTCTGCCCTGCTCTCC	1224-1203	343
Itga3	NM_013565	TGGCCGTGGGTGCTGGGTTGC	3374-3394	AAGGGAGCGCCGGAAGGTTTGTCG	3760-3737	387
Itga4	NM_010576	CAACCGGGCACTCCTACAACCTG	321-343	AACACGGGGCCATCCTTTTACTCA	785-762	465
Itga5	NM_010577	TCGGGAGGACTGCAGGGAGATGAA	1987-2010	GAACCGAAGGCCACCCCAGAGACT	2494-2471	508
Itga6	NM_008397	GGGGCACCTCGGGCAGAAGCACT	249-271	CAACCGGCCATCGCAGAAACTCCA	593-570	345
Itga7	NM_008398	GGAGGGCGAGGCGGTGGGTAT	1463-1483	ACGGTGCCGGAGGACTGATGC	1858-1838	396
Itga8	NM_001001309	TGAAGCAGCAGAAATCCCTCCACT	1850-1873	TGCGCTCAATCCCAACATAATCTG	2245-2222	396
Itga9	NM_133721	TGGCCCACGTCAAGCGGAGAG	1898-1918	CAGCCCCCAAAGCCAGGTGTG	2176-2156	279
Itga10	NM_001081053	GGTGGACGCCGCCTCTTTCTCT	1375-1396	TGCCCCCACCGCTACATCA	1764-1746	390
Itga11	NM_176922	AACGGGGCGGTGCTGAAGGAGACA	1255-1278	CAGAGGCGATGCAGGACCCGAACC	1729-1706	475
Itgad	NM_001029872	GGGCTCCAGCTTGCAGTTCATCC	408-430	TTTTTCGGGCCCCATTCTTGCTAT	769-746	362
Itgae	NM_008399	GCCGTGGCTCCAGAAAGAAAG	913-933	AGCGCACCCCCAGACCAGTTA	1318-1298	406
Itgal	NM_008400	GATTGCGGGCCCCAGACTTT	133-152	TGATAGGCGGGACGATTTTGTAAC	555-532	423
Itgam	NM_001082960	GCCGCCCCAGCAGTTCC	499-515	GGGCCCCATTGGTTTTCTGA	839-820	341
Itgav	NM_008402	CGCCCCGCCGCCCTTGACC	204-222	GCTGGCACCGGCGGCTGGATG	633-613	430
Itgax	NM_021334	GCTTTGGGGCGGCTCTGACA	1614-1633	GGGGCCACCTGCTCCTGACA	1989-1970	376
Itgb1	NM_010578	TGGGCACACTGTCTGGAAACTCTA	1249-1272	CACTTGGGACTGGCTGGGATGC	1609-1588	361
Itgb2	NM_008404	GGCCCTGACCCACCTGACTGA	2290-2310	TTGGGGCCACCTTTACTGAGACTG	2712-2689	423
Itgb3	NM_016780	GCTGGCGGGCGTTGTTGTT	143-161	GATTGCCTGTGGTGGGGAGATGTA	683-660	541
Itgb4	NM_001005608	ACCGGGGTCGCTGTTCTATGG	2104-2124	CCGGGAGGGCAGTCTTTCTTTT	2569-2548	466
Itgb5	NM_010580	ATCCGGAATGGCTGTGAAGGTGAG	412-435	TCGGTGCCGTGTAGGAGAAAGGAG	787-764	376
Itgb6	NM_021359	CCAGGCCCAAGAAGAGTGTGCT	2117-2138	TCTGGCCGAAGGAGGTCTGTCTA	2584-262	468
Itgb7	NM_013566	ACCTTGGCCCCTAACTTGGATGAT	2120-2143	GACGCTGCCCTGCCCTTCTAT	2561-2541	442
Itgb8	NM_177290	CTTGGGGCAGCCTGGGTATTTTCA	557-580	GATCCACGGGGTATTTCTTCAG	923-902	367
ephrin A1	NM_010107	TCTGCCCACATTACGAGGACGACT	248-271	GGGGGCGGCACTGTAACCAAT	654-634	407
ephrin A2	NM_007909	CCGCCGCCGCCTGGTTTCTCG	99-119	GGCGCCCCGTAGTGTGG	505-489	407
ephrin A3	NM_010108	GGCGGCGGGGCGGAGCAGTA	260-279	CGATGTGGAGGCGCAGCAGACGAA	535-512	276
ephrin A4	NM_007910	TCGCCGGGACAGAGAAGTG	17-35	CAGCGCTGGAAGGCATTT	368-350	352
ephrin A5	NM_207654	CGCCCCTTCATCGTATTTATTCCT	56-79	GGCCGGTTACATTCCCATCTCTTG	501-478	446
ephrin B1	NM_010110	CCAGCCGGCCAAGCAAGGAGTCA	1289-1311	GCCGCCCGCTGCTGTGTATGCT	1586-1565	298
ephrin B2	NM_010111	TGTTGGGGACTTTTGATGGTTTTG	202-225	AGCAGCGGGGTATTCTCCTTCTTA	464-441	263
ephrin B3	NM_007911	CGGGGAGCAAGCATCGCAGAAAAG	211-234	CGAGCCCGGGGACAAAGTAGAT	564-543	354
Eph Receptor A1	NM_023580	AGCAGCGCCTCCCTCAGTATCAAC	1361-1384	CACGCTGCCTCTGCTGTCTCTGTC	1808-1785	448
Eph Receptor A2	NM_010139	AGCCCCCGACTCCCTCAAGACG	2744-2765	GCACGGCCCCAATGGACTCAGATA	3064-3041	321
Eph Receptor A3	NM_010140	GCCTACCGCAAGTTCACCTCAG	2502-2523	CCTCATGCCGTTAAGCCAATCAC	2885-2863	384

Eph Receptor A4	NM_007936	GCCGCCGGGTACACGACACT	2857-2876	AAGCCCCATGGTTTCAGCAATCTC	3204-3181	348
Eph Receptor A5	NM_007937	AAAGGGGAAGATTGCAAAGAACAG	1819-1842	AGAAGCCCTCCCACAGCCACAATC	2215-2192	397
Eph Receptor A6	NM_007938	ACTTCATGGCAGCGGGTTTTACA	3507-3529	TGCAGTGGGCCAGAGTTTCGTT	3899-3878	393
Eph Receptor A7						
variant 1	NM_010141	GTGGCCGCAGGTTCTACAAATCT	1068-1090	TTCCCAACTGCACCGCTTACAC	1357-1336	290
variant 2	NM_001122889	CTGACAACGGGGGAAGAAACGAT	1303-1325	TATTAATGGAGGCGGAGGTGGACT	1749-1726	447
Eph Receptor A8	NM_007939	CGGGGCGCGGCGAAGTGAA	146-164	CCCAGGTCTGCGCCCGTGAAGC	545-524	400
Eph Receptor A10	NM_177671	CACCCGCTGCGCCTGTTCGTCT	112-133	CGGCCCAGGTCGGTCTCGGTTTCT	506-483	395
Eph Receptor B1	NM_173447	CTGCCCCCTCCACTGTTCCTATCA	1589-1612	GCCTCGGCCTGTGCTGTAATGTTG	2055-2032	467
Eph Receptor B2	NM_010142	CCAACGCCGGCTTTACCTCTTTC	2909-2931	CCCCCACCCAATCCCCTTTTC	3267-3247	359
Eph Receptor B3	NM_010143	GGCCGCCGGGAGGTGTTC	2326-2343	AAGCGGGAGAGGCCAAAGTCAGAG	2705-2682	380
Eph Receptor B4	NM_010144	ATACGCCCGCCTGGATGGAGAACC	818-841	CGCGCAGCTGCAGCCCGTGAC	1209-1189	392
Eph Receptor B6	NM_007680	CTGGAGGCCGGCGGGAAGGTT	236-256	GCGGGTAGGTGAGCCAGCCAATCT	582-559	347
Semaphorin 3A	NM_009152	TCATGGGACGGGACTTCGCTATC	1230-1252	CATTGGGACCGGGCACAGA	1547-1529	318
Semaphorin 3B						
variant 1	NM_001042779	AAGGGAAGGGCAAGAGGCAAGGAG	74-97	GGCGGGGAAGGTTAGGGGCAGTAT	490-467	417
variant 2	NM_009153	GCCGAGCCTTCTTGGGACCTTTTG	1252-1275	TCGGCTGCCTTTGGGGACTGAGAT	1613-1590	362
Semaphorin 3C	NM_013657	CCCACCCTCCGGACTCACTAAG	37-58	ATGCTGGCCAGAAAACACTCAAAG	468-445	432
Semaphorin 3D	NM_028882	GGTGGGCAACGAAGTCTGATAAAC	1027-1050	CCTGCCACTGGGTACACGGACTT	1463-1441	437
Semaphorin 3E	NM_011348	GGCCGAGCACAGCAGTAAAGGTAG	918-941	CGCCGAGTCTCTGCCCCAATAG	1237-1216	320
Semaphorin 3F	NM_011349	TTGCCCGCGATCCCTACTGTG	1787-1807	CCGCGTGACGATGTGCTTGAA	2181-2161	395
Semaphorin 3G	NM_001025379	TCTACCAACCGCTCTGCCATCTTT	162-185	CAACGCCCCCTTCCATTCTCG	547-527	386
Semaphorin 4A	NM_013658	CTCCATGGGCCCCTCCTCTGACA	1298-1320	CTGCAATTGGCCCTGGGAACTCTC	1654-1631	357
Semaphorin 4B	NM_013659	CCCGAGTTGCCCGAGTCTGTAAGG	1085-1108	CGTTCCCGGGCACTGTTGGTAAT	1466-1444	382
Semaphorin 4C	NM_001126047	CCCTCGGCCTGGTTCGTGTATC	1495-1516	TGAGAGCGGGAGCCAGCAAAGAG	1875-1853	381
Semaphorin 4D	NM_013660	CCCGCCCACAGCTACACATCAGTC	908-931	CCGGGGCCCTCAGCACAAAC	1337-1318	430
Semaphorin 4F	NM_011350	TGGGGGCTTATGCTTGCGAGTGTC	1964-1987	CAGGGGCAGCCGTTCATCTTCAGG	2283-2260	320
variant 2	NM_001113481	CGGTTTGCGGCCCCTCATACG	311-331	ACATTTCCCCCGGCCACTCTCAAG	655-632	345
Semaphorin 4G	NM_011976	GTGCCCGTGACATAAGAGATAGGA	630-653	CGGGCCGGGTCATAAGGA	897-880	268
Semaphorin 5A	NM_009154	GAGAGCCGGCCTTGTGTATTTGA	3464-3486	GGGCAGCCATGGAGCACGGTTTT	3948-3926	485
Sempahorin 5B	NM_013661	ATCGGGCTGTTTGCGTATTTCTTC	994-1017	AGCCCTGGGGTTTTCCTGGTA	1350-1330	357
Semaphorin 6A	NM_018744	CACCCGCCGTCCGAGCAGTC	6114-6133	GCGGGGTGTGGGGTGTAGGAATGT	6522-6499	409
Semaphorin 6B	NM_013662	CGCCCGGCCCTGTCGTTTTT	298-317	ATTGGCGCAGATGGGATTGAA	738-718	441
Semaphorin 6C	NM_011351	GCTGCGGAACTTGGGCTGGACTTT	188-211	GCTGCGGCACATAGGGCTGAA	472-452	285
Semaphorin 6D						
variant 1	NM_172537	GTGGCCTTCTCTAGCTGCGTGGTC	2320-2343	ACTGGGCGGATTCTGCGTCTTTA	2749-2727	430
variant 4	NM_199241	GGCCTTCTCTAGCTGCGTGGTC	1503-1524	ATTTCCGGGAGCTCGTCAGTTTAG	1870-1847	368
variant 6	NM_199240	GGCCTTCTCTAGCTGCGTGGTC	1503-1524	ATTTCCGGGAGCTCGTCAGTTTAG	1813-1790	311
variant 2	NM_199238	GCAGCGCCGAGAGTGAGGAG	1430-1449	ATGAACGCGCCCAAGACAAAAG	1808-1787	379
variant 5	NM_199239	GGTTAAGCCAGGGAGTTTGTGAG	1604-1626	ACTGGGCGGATTCTGCGTCTTTA	1987-1965	384
Semaphorin 7A	NM_011352	CGGCTGCGGCTTCTGCTGGTGTT	104-126	CATCCGGGCTGAAGGGGGCATAG	536-514	433
Plexin A1	NM_008881	CCTCCCGTGGGCCTCTGTCTGG	3161-3182	CGGGCTGCGCTTTGGGTTGTCTAT	3606-3583	446
Plexin A2	NM_008882	GCGCGGGCCGAGGATTTT	415-432	ACGATTGATAGCCCCCACATACAC	850-827	436
Plexin A3	NM_008883	GGCGGCGGTTGCAGATTTTG	12-31	ACATGGGCCCGTAGCTCAGTTAGG	380-357	369

Plexin A4	NM_175750	TCTGATTTGGCCCCCTCCGTTTTT	683-706	TCCTCGCAGACACAGCAGCACTC	962-940	280
Plexin B1	NM_172775	GCCCGGGAAGCCTGTAATGA	3264-3283	CACGGGCTGGGAAGATGGAGT	3621-3601	358
Plexin B2	XM_001003435	CGGCGGGCCCAAGGAGAGGT	1833-1852	CCAAAGGGTCAGGGGCCAGGAACT	2268-2245	436
Plexin B3	NM_019587	GACACCCAAGGCCAGCTACATAAG	1266-1289	GGGCAGCCGAGGAACAGACAGAAT	1676-1653	411
Plexin C1	NM_018797	ACGGGGCTGTGCAGGTCTTCTAC	1973-1995	GTTATGTTTTGCCCACCGCTGAT	2324-2302	352
Plexin D1	NM_026376	CCCCGCTGGCTGTCAAGTATTTCT	5398-5421	CTCGGCCAGGTGTGCGTTCAT	5777-5757	380
Dcc	NM_007831	GCTATCCTCCGCCAAGTTTCACC	1417-1439	GACCACCCCAAGGATCCGTAAGTT	1796-1773	380
NGFR	NM_033217	GTGTGCGGGGTGGGCTCAG	528-546	CCTCCGGGGGCGTAGACCT	759-741	232
NRP1	NM_008737	TCGTCCGCGCAATCGTGTTC	291-310	CCGCCTTCATTCTCCCCATCA	637-617	347
NRP2	NM_001077403	GCAGCGCTAACCCCAATGACAG	2900-2921	GATAATGCGCCCGTGCTTCC	3168-3149	269
Robo1	NM_019412	CGGCCTCCCCCAGTCATTC	2228-2246	AGTTGGGGTCGGTGGGTCTTG	2549-2529	322
Robo2	NM_175549	CCAAGTGCCCCTCCCCAGTC	2206-2225	GCTATAAATGCCGGTTGCTTCACG	2588-2565	383
Robo3	NM_001476840	CCCAGGGGCCCAAATCCA	2450-2467	CACGCCCACGCCAGCACT	2810-2793	361
Unc5c	NM_009472	TGGCGCAGGAGGGAGTG	1153-1169	GGGGGACAGCCAGGAGAT	1468-1451	316
