# Supplementary material for: 3D Timelapse Analysis of Muscle Satellite Cell Motility
Source: Stem Cells. 2009 Oct;27(10):2527–38. doi: 10.1002/stem.178 (PMC2798070; doi:10.1002/stem.178)
Supplement: Supplementary file 2 [file stem0027-2527-SD2.pdf]

Movie #	Track #	Persistent association of sister cells	Comigration of sister cells	interaction with unrelated cell	Comigration of unrelated cells	Individual Spinning	Extensive interactions	Projection into the matrix	Motility within the matrix
1	1	x	x				x		
1	2								
1	3								
2	1								x
2	2							x	
3	1							x	
4	1	x	x				x	x	
4	2	x					x		
5	1						x	x	
5	2					x			
6	1						x		
6	2					x			
6	3	x					x		
6	4								
7	1							x	x
7	2	x	x						
8	1					x			
9	1		x						
9	2			x					
9	3			x				x	
9	4		x	x				x	
9	5	x				x	x		x
10	1	x				x	x		
10	2	x				x	x		
10	3								
11	1		x	x	x			x	
11	2					x			
12	1	x	x				x		
12	2								
13	1			x					
13	2							x	
13	3		x						
14	1	x							
15	1					x		x	
16	1								
16	2							x	
17	1					x			
17	2	x	x			x	x	x	
18	1	x				x	x		
18	2	x				x			
19	1	x	x				x		
19	2			x			x		
19	3								
19	4	x		x	x		x		
20	1								
21	1	x							
21	2			x	x				
21	3			x					
22	1		x				x		
23	1	x	x						
24	1		x						
25	1								
26	1								
26	2					x			
26	3								
27	1	x					x		
27	2	x					x		
27	3								
28	1					x			
28	2							x	
28	3	x		x			x		
29	1	x					x		
30	1								
30	2								
31	1			x			x		
31	2	x				x	x		
31	3	x		x	x		x		

31	4	x	x	x		x		
31	5			x			x	
32	1							
33	1	x					x	
33	2			x		x	x	
33	3	x		x			x	
34	1							
35	1	x						
35	2	x				x	x	
36	1							
36	2					x		
36	3	x				x	x	
37	1	x					x	
38	1							x
38	2	x	x				x	x
38	3	x	x					x
38	4	x					x	x
39	1		x	x			x	
39	2					x		
39	3	x		x	x		x	x
40	1					x		x
41	1	x	x					
41	2							
42	1	x	x			x	x	
43	1			x		x	x	
43	2	x	x					x
43	3	x				x		x
44	1			x			x	
44	2			x	x			x
45	1					x		
45	2							
45	3							
46	1							
46	2	x	x			x	x	x
46	3	x					x	x
47	1					x		x
47	2	x					x	
47	3	x						
48	1			x		x		
48	2			x				
48	3	x	x				x	x
49	1					x		
49	2	x		x				x
49	3	x	x					
49	4							
50	1	x				x	x	
51	1			x				
51	2					x		x
52	1					x		
52	2							x
53	1					x		
54	1	x	x					x
55	1	x	x				x	
55	2							
56	1							
56	2							x
57	1							x
57	2	x					x	
57	3	x	x			x		
57	4			x				
58	1							
58	2			x		x		
58	3			x				
58	4			x				
59	1							
60	1		x				x	
60	2							x
61	1	x		x			x	
61	2	x	x	x		x	x	
61	3	x	x			x	x	
61	4	x	x			x		

62	1			x			x		
62	2		x	x	x		x		
62	3			x			x	x	
63	1	x	x	x		x	x		
63	2			x		x			
63	3	x	x			x		x	
64	1		x			x			
65	1								
66	1								
66	2			x		x		x	
66	3	x		x		x	x	x	
66	4	x				x		x	x
67	1			x				x	
68	1					x			
68	2					x		x	
68	3					x			
69	1			x				x	
69	2			x			x	x	
70	1	x	x			x	x		
70	2								
70	3	x	x	x		x	x		
70	4	x		x	x		x		
71	1								
71	2	x				x			
72	1	x					x	x	
72	2	x				x	x		
73	1	x	x			x	x	x	
73	2	x	x			x	x	x	
74	1	x		x				x	
75	1	x						x	
76	1	x	x	x	x		x		
76	2	x	x	x	x	x	x		
77	1	x	x				x	x	
78	1			x			x		
78	2			x	x		x		
78	3	x	x	x	x	x	x		
79	1	x				x	x		
79	2					x			
79	3					x			
80	1			x	x			x	
81	1	x	x	x		x	x		
82	1	x	x			x		x	
83	1	x	x	x					
84	1			x		x		x	
84	2			x				x	
84	3			x		x			
85	1								
85	2					x			
86	1					x			
86	2					x			
87	1			x		x	x		
87	2	x	x	x			x	x	
87	3	x	x	x		x			
87	4	x	x	x	x	x	x		
88	1	x	x	x	x	x	x	x	
88	2			x	x		x	x	
89	1	x	x	x	x	x	x		
89	2	x	x	x	x		x		
89	3	x	x			x	x		
89	4	x	x			x		x	
90	1	x	x	x		x	x	x	
90	2	x	x			x	x	x	x
91	1	x				x		x	
91	2					x	x	x	
92	1								
92	2								
93	1					x		x	
94	1	x	x			x	x		
95	1						x	x	
95	2					x			
96	1	x				x	x	x	

96	2	x	x	x			x	x	
97	1								
97	2								
97	3								
97	4								
98	1	x				x	x	x	
99	1							x	
99	2	x	x	x		x	x		
100	1	x	x			x	x	x	
100	2	x				x	x	x	
101	1								
102	1					x		x	
103	1	x	x	x			x	x	
103	2	x					x		
103	3	x	x	x			x	x	
103	4			x			x	x	
104	1	x	x				x		
104	2	x		x	x				
104	3			x	x		x	x	
104	4	x				x	x	x	
105	1	x	x	x			x		
106	1					x		x	
107	1	x	x				x		
108	1	x	x	x			x	x	x
108	2			x				x	
109	1			x	x		x	x	x
109	2			x		x		x	x
109	3								
110	1		x			x	x		
111	1			x		x			
111	2	x	x			x	x	x	
111	3	x	x	x				x	x
112	1	x		x		x	x		
112	2					x	x		
113	1	x	x	x	x		x	x	
113	2	x		x		x	x	x	x
113	3	x	x	x			x	x	x
114	1	x	x	x		x	x		
114	2	x		x	x	x	x		
114	3			x					
114	4			x	x		x	x	x
115	1	x	x	x	x		x	x	
115	2	x	x				x	x	
115	3	x	x	x	x	x	x	x	
115	4	x	x	x	x		x	x	
115	5					x		x	
116	1	x	x				x		
116	2	x	x				x	x	
116	3			x			x	x	
117	1			x		x		x	
117	2					x			
118	1	x	x			x		x	
118	2			x				x	
118	3			x	x	x	x		
119	1	x	x	x	x	x	x		
119	2			x	x		x		
119	3			x			x		
120	1	x	x			x	x	x	
120	2	x	x			x		x	
121	1	x				x		x	
	Total #	122.00	81.00	96.00	30.00	108.00	121.00	98.00	12.00
	%age	45.35	30.11	35.69	11.15	40.15	44.98	36.43	4.46
