# Supplementary material for: 3D Timelapse Analysis of Muscle Satellite Cell Motility
Source: Stem Cells. 2009 Oct;27(10):2527–38. doi: 10.1002/stem.178 (PMC2798070; doi:10.1002/stem.178)
Supplement: Supplementary file 3 [file stem0027-2527-SD3.doc]

**SUPPLEMENTAL MOVIE INFORMATION**

Movies are supplied as Quicktime; if you do not have Quicktime free downloads are available at<http://www.apple.com/quicktime/download/> . To play Quicktime movies, click the Play button (▶); to advance the movie one frame at a time click on the ▶▶ button; the slider at the bottom of the movie (▼) can also be used to advance and rewind as desired. To change the movie size select “View” from the menu bar and select the desired frame size. To change the playback speed of the movie, select “Window” from the menu bar then “Show A/V Controls”, playback speed can be altered from ½ speed to 3x speed via the slider at the bottom right.

**Details of movies showing satellite cell ‘behaviors’ discussed in the text:**

**Movie 4a1:** inclusive time 24 hours to 48 hours after activation, stills in text taken at 28:30; 28:40; 30:10; 32:10; 33:20; and 45:40 hours. A single cell (originally on the lower side of the fiber at the upper right of the screen) travels by pseudopodial motion to the end of the fiber, pauses, returns up the fiber, and divides approximately ¾ of the way into the movie. The two daughter cells remain physically associated and move down the fiber, then rapidly dissociate and travel away from one another. They divide within 10 minutes of one another, and one set of daughter cells remains closely associated while the other dissociates and moves apart.

**Movie 4a2:** inclusive time 24 hours to 51 hours after activation, stills in text taken at 30:00; 30:30; 32:20; 34:30; 35:20; and 48:50 hours. A single cell located on the lower side of the fiber divides in place without previous movement, then the daughter cells begin movement in the region near the original cell. The daughter cells divide within 10 minutes of one another, and both pairs of daughter cells remain associated for at least two hours. One pair dissociates then reassociates briefly while the other remains closely associated; one daughter from each set divides within 10 minutes of each other, followed by the other associated cell (on the left.) The four cells in this cluster remain associated for an hour, following which one cell dissociates from the other three and moves away.

**Movie** **4b:** inclusive time 12 hours to 36 hours after activation, stills taken at 21:40; 25:20; 25:50; 26:30; 29:20; and 36:00 hours. Beginning at 12 hours (prior to any cell division) one cell located towards the center of the fiber on the bottom left (in what appears to be a lamina ‘divot’) and a cell located on the lower edge of the fiber approximately 2/3 of the way up the fiber begine to move on the surface of the fiber, circle around, make contact with one another via membrane extensions and then become very closely associated for the remainder of the movie. Based on cases in which other cells exhibiting such close interactions later dissociate into two motile cells, or live cell tagging studies in which a cell with labeled plasma membrane is very closely associated with an unlabeled cell which remains unlabeled until the cells dissociate, we do not believe that this represents a potential fusion event (see next movie for an addition example.)

**Movie 4c:** inclusive time 48 hours to 72 hours after activation, stills taken at 48:00; 54:20; 59:20; 61:30; 69:10; and 72:00 hours. Two sets of satellite cells remain very closely associated for an extended time, interacting extensively but not fusing. Cells in the top group in particular will occasionally move on top of the other cell(s), then back down onto the fiber.

**Movie 4d:** inclusive time 48 to 72 hours after activation; stills taken at 52:20; 63:20; 65:20; 66:40; 67:10; 68:00 hours. At least one cell at the right edge of a bundle of fibers repeatedly extends protrusions into the collagen matrix, then moves off the fiber; another cell moves out along the first cell, divides, and the daughter cells continue in opposite directions. Note that another fiber, running roughly parallel to the bundle, is present and can be seen in the top right corner of the image. While we cannot conclusively determine myogenic identity for these cells or the cell in Movie 4e, and the morphology shown by cells moving through the collagen matrix is frequently different from the same cells or other cells while adhered to the myofiber, in our hands all motile cells in preparations from myogenic lineage factor-tagged transgenic mice are marked as myoblasts.

**Movie 4e:** inclusive time 62 hours to 70 hours after activation, stills taken at 64:00; 65:10; 66:50; 67:20; 68:00; and 68:50 hours. A cell located in the collagen matrix at the start of the movie moves by pseudopodial motion parallel to a fiber, turns, contacts the fiber and apparently becomes adherent, taking on a more rounded morphology. Note: area of magnified image in still figures is moved from frame to frame.

**ADDITIONAL MOVIES DOWNLOADABLE FROM** http://cornelisond.biology.missouri.edu/Cornelison_lab/movies/index.html

To generate Supplemental Table 2 and Figure 4f, 120 movies containing 24-48 hour timeframes of 270 tracked FGF2-treated cells were scored for occurrence of 8 specific activities:

Persistent association = Two cells must have arisen from a cell division in the movie, then stay in contact with each others' membrane for at least 1 hour.

Comigration of sister cells = Two cells must have arisen from a cell division in the movie, then move incrementally together and follow the same track for at least an hour.

Interaction with unrelated cell = Two cells that did not result from the same cell division and are originally not in contact with each other establish cell-cell membrane contact for any duration.

Comigration of unrelated cells= Two cells that did not result from the same cell division and are originally not in contact with each other maintain cell-cell membrane contact, move incrementally together and follow the same track for at least an hour.

Individual spinning = A single cell oscillates in place, sampling the basal lamina of the fiber, for an extended period (at least 4 hours.)

Extensive interactions = Two cells (usually related) circle over and around one another, maintaining very close apposition and cell-cell membrane contact, for extended periods (at least 4 hours.)

Projection into the matrix = A cell extends a visible projection out into the matrix.

Motility within the matrix = A cell completely leaves the fiber membrane for any length of time.
